# Egg retrieval in ground-nesting cuckoo hosts: can two species of buntings accurately identify and retrieve their own eggs?

**DOI:** 10.1007/s10071-024-01919-w

**Published:** 2024-12-12

**Authors:** Yuhan Zhang, Guo Zhong, Longwu Wang, Wei Liang

**Affiliations:** 1 Ministry of Education Key Laboratory for Ecology of Tropical Islands, College of Life Sciences, Hainan Normal University, Haikou, 571158 China; 2 School of Life Sciences, Guizhou Normal University, Guiyang, 550001 China

**Keywords:** Egg retrieval, Brood parasitism, Egg recognition, Ground-nesting birds, Buntings

## Abstract

Egg retrieval in birds may help ensure the survival of eggs and improve reproductive success. However, with the risk of brood parasitism, for ground-nesting or cavity-nesting bird hosts, there is a significant reproductive cost and thus a reduction in fitness if the host wrongly retrieved the parasitic eggs. The south rock bunting *(Emberiza yunnanensis*) and yellow-throated bunting (*E. elegans*) are hosts for common cuckoos (*Cuculus canorus*), which coexist within the study area and breed sympatrically in ground nests. Previous studies have found that these two species exhibit strong egg recognition and egg rejection of non-mimetic eggs. In this study, red model eggs, budgerigar eggs, and the host’s own eggs were used to assess the recognition and retrieval behavior of two bunting hosts, particularly in response to different types of eggs placed at the nest edge. The results showed that both bunting hosts retrieved ca. 80% of own eggs and did not retrieve any red model eggs. This indicated that both species could distinguish non-mimetic model eggs from their own eggs and make appropriate decisions, which is consistent with their responses when encountering foreign eggs in the nest. However, both species simultaneously retrieved some (8.3% for the yellow-throated bunting and 19% for south rock bunting) of the highly mimetic budgerigar eggs, indicating that the degree of mimicry of foreign eggs affects their egg recognition and egg retrieval behavior. Factors such as parasitism risk, nest predation pressure, age differences, and experience of parent birds may combine to influence egg retrieval behavior of the host.

**Supplementary Information:**

The online version contains supplementary material available at 10.1007/s10071-024-01919-w.

Egg retrieval behavior by birds is the act of the parent bird retrieving and incubating eggs that have rolled out near the nest. This behavior is beneficial for the integrity of the clutch and improves reproductive efficiency (Lorenz and Tinbergen 1938; Lank et al. 1991; Spena et al. 2020). For most nests, such as those built by birds in trees, eggs that have fallen out will break, thus egg retrieval behavior is impossible. However, for ground-nesting and cavity-nesting birds, some displaced eggs stay at the nest edge without breaking, so retrieval behavior is beneficial for offspring survival (Lank et al. 1991; Zaun and Weathers 2009).

Previous studies have found that most birds exhibiting egg-retrieval behavior are ground-nesting precocial birds, such as snow geese (*Anser caerulescens*) (Lank et al. 1991), Eurasian stone curlews (*Burhinus oedicnemus*) (Spena et al. 2020), and American coots (*Fulica americana*) (Lyon and Shizuka 2017). Precocial birds have simple nest structures, and due to their larger body, they are prone to easily eject eggs from the nest when startled or during nest departure. Therefore, egg retrieval behavior in these birds is necessary and potentially consequential to reproductive success. The nests of passerine birds are more complex and denser compared to other groups of birds, providing good containment for the eggs in the nest. However, studies have found that some hole-nesting passerines, such as tits (Paridae), also possess strong egg retrieval abilities (Yang et al. 2019; Liu et al. 2023).

Avian brood parasitism may be an important factor contributing to the evolution of egg-retrieval behavior in passerines (Yang et al. 2019, Yang and Zhang 2024). Brood parasitism is a specific reproductive behavior in which some birds do not build nests during breeding, but instead lay their eggs in the nests of other birds for them to incubate and raise the offspring, with the parasitized hosts often incurring significant reproductive costs (Davies 2000; Feeney et al. 2014; Soler 2017). Some exclusionary cuckoos, e.g., the common cuckoo (*Cuculus canorus*), have parasitic chicks that push the host’s offspring out of the nest (Davies 2000; Stokke et al. 2005). Therefore, the host’s retrieval of eggs or chicks at the edge of the nest is, to some extent, an anti-parasitic measure that can reduce breeding losses. However, the host’s indiscriminate retrieval of all eggs at the edge of the nest may also lead to a decrease in its fitness. Parasites may lay eggs at the edge of the host’s nest due to pressure from the host’s nest defense, or in the confined space of small cavity nests (Rutila et al. 2002; Avilés et al. 2005). For example, Samaš et al. (2016) recorded that approximately half of the parasitized eggs of the common cuckoo were laid at the edge of the host’s nest, the common redstart *(Phoenicurus phoenicurus*). Studies have revealed that some parent birds may not only retrieve their eggs but may also retrieve egg-shaped stones or other non-egg-shaped objects at the edge of the nest in error (Lorenz and Tinbergen 1938; Langlois et al. 2012; Lyon and Shizuka 2017). If the host retrieves parasitic eggs by mistake from the edge of the nest, it will do so at great reproductive cost to itself.

Recognition and rejection of parasitized eggs is an effective anti-parasitism strategy of the host (Rothstein 1990; Moksnes et al. 1991; Antonov et al. 2011; Soler 2017). Both egg rejection and egg retrieval have important impacts on the reproductive success of cuckoo hosts (Shettleworth, 2010), but these two behaviors have opposing motivations and appear to conflict with each other. Both behaviors involve egg recognition and cognition by the parent bird (Conover 1985; Yang et al. 2019). The visual cognition utilized by parent birds of the same species during egg retrieval and egg rejection, and the relationship between the two behaviors, was first examined by Lyon and Shizuka (2017). In a comparative study of egg retrieval over egg rejection in American coots, which are precocial birds, coots were observed to use different cues for the two behaviors. In contrast, Yang et al. (2019) found that the green-backed tit *(Parus monticolus*) exhibited varied retrieval and rejection of experimental eggs according to the degree of egg mimicry. A further study on the green-backed tit and Japanese tit (*P. minor*) showed that egg spots are an important clue for egg retrieval, and these birds consistently reject non-mimetic eggs both at the edge and inside the nest (Liu et al. 2023). Past studies have shown that differences in egg location affect parental cognition and behavior and that interrelationships between egg retrieval and egg rejection behaviors vary among species and even different populations of the same species (Yang and Zhang 2024). Therefore, the study of egg retrieval behavior in a wider range of hosts could help to explore the relationship between egg retrieval and egg rejection behavior.

Buntings are suitable hosts for cuckoos but they are rarely found to be parasitized (Wyllie 1981; Moksnes and Røskaft 1995; Yang et al. 2012). Studying the behavior of bunting hosts in recognizing and retrieving eggs can help uncover the reasons behind the rarity of brood parasitism and facilitate the exploration of potential host-parasite relationships. The South rock bunting *(Emberiza yunnanensis*) and the yellow-throated bunting (*E. elegans*) belong to the same genus in the family Emberizidae. They construct nests in open ground and breed sympatrically within the study area. They are potential hosts for common cuckoos in previous studies and have a strong ability to recognize and reject red non-mimetic eggs from the nest (Zhang et al. 2023). However, there have been no studies on the egg retrieval behavior of these two species. The eggs of the two species are white or grayish-white in color with black or brown speckles (Ding et al. 2019; Zhang et al. 2023). Red model eggs were completely non-mimetic in terms of egg phenotype and material in comparison to the host eggs. Budgerigar (*Melopsittacus undulatus*) eggs were pure white in color, similar in color to the host’s eggs. Budgerigar eggs demonstrated a higher degree of mimicry compared to the red model egg but were also non-mimetic. In this study, we used red model eggs, budgerigar eggs, and the host own eggs to investigate the ability of the two species of buntings to retrieve eggs and to test whether they could accurately recognize and differentiate between different types of eggs at the edge of the nest and make the “correct” decision. We hypothesized that, for both bunting species, the majority of individuals would opt to reject non-mimetic eggs at the nest edge, whereas they would consider retrieving their own eggs.

## Methods

### Study area and species

The experiment was carried out in Liuzhi area (26°10’ − 14’ N, 105°13’ − 24’ E), Guizhou Province, southwestern China. The average altitude of this area is between 1,200 and 1,400 m, with an average annual temperature of 14.5 ℃. This area has a subtropical monsoon climate with a karst mountain landscape type consisting of rivers, arable land, villages, and forests (Zhang et al. 2023).

We searched for nests and conducted fieldwork at this site during the breeding season (April to August) from 2020 to 2023. Both the yellow-throated bunting and south rock bunting are distributed in Guizhou and build nests on open ground in the study area (Zheng 2023; Zhang et al. 2023). Yellow-throated bunting eggs are characterized by a white or grayish-white base color with black or brown spots, and the number of eggs per clutch ranges from three to six (Ding et al. 2019). The South rock bunting was previously considered a subspecies of Godlewski’s bunting (*Emberiza godlewskii*) (Li et al. 2023). Its eggs are characterized by a white or grayish-white base color with black or brown spot-like, rod-like, and filamentous patterns, with the number of eggs per clutch ranging from three to five (Ding et al. 2019).

### Egg retrieval experiments

In the study area, nests of the two bunting hosts were hidden under thickets or grasses, and also on soil ridges and between stone gaps along cornfields (Zhang et al. 2023). Finding the experimental nests was based on nest site characteristics of ground nests of the two species of buntings. Each nest was numbered and the GPS location was recorded. A total of 33 nests of yellow-throated buntings and 51 nests of south rock buntings were found and subjected to egg retrieval experiments during the experimental period, with at least 50 m between nests of the same species.

**Fig. 1:**
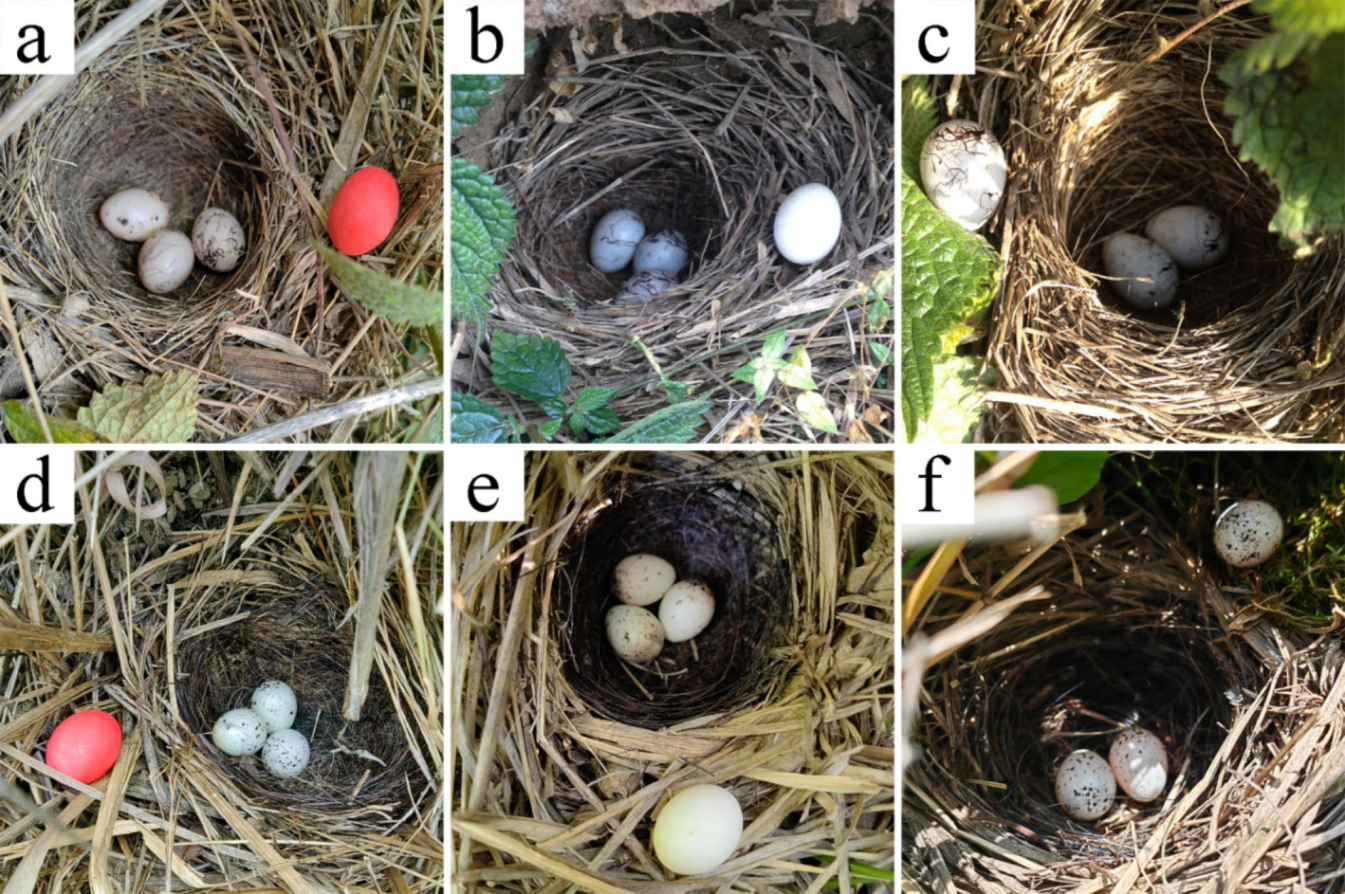
Photographs of egg retrieval experiments conducted in the nests of south rock buntings and yellow-throated buntings (*a*, *b*, and *c* are the responses of south rock buntings to a red model egg, a budgerigar egg, and their own egg placed at the rim of the nest cup, respectively. *d*, *e*, and *f* are the responses of yellow-throated buntings to a red model egg, a budgerigar egg, and their own egg placed at the rim of the nest cup, respectively)

Experiments were conducted during the early stages of egg incubation of the host, and experimental eggs were placed 2 to 3 cm from the inner edge of the nest cup (Fig. 1). The following sets of manipulations were set up: (1) placing one red model egg made of red clay and sized according to the host’s eggs (Zhang et al. 2023), (2) placing one budgerigar egg similar in size to the host’s eggs, and (3) removing one host egg from the host nest and placing it at the edge of the nest (Fig. 1). Previous studies have reported that a few individuals in the early stages of egg incubation begin to lay additional eggs within 1 to 3 days of incubating the initial clutch (Ding et al. 2019). To address this, we used a small marker pen to mark the tips of the eggs that were removed with dots, to determine whether the eggs in the nests were experimental eggs that were retrieved by the hosts or eggs that were laid later in the nests.

All nests were checked on the second day of the manipulations, then checked on the third and fifth day, for a 6-day experimental cycle. Eggs were recorded as retrieved if they were inside the nest cup, undamaged, or without peck marks; rejected if they disappeared or had peck marks; and neglected if they remained at the nest edge, with no change in position and without any peck marks. For the experimental nests found, one of the three manipulations was randomly selected for the experiment, with a sample size of no less than 10 nests for per condition group. Only one experiment could be performed for each nest.

### Data analysis

For differences in egg rejection and retrieval rates between species and between experimental groups, differences were tested for statistical significance using the Chi-square test and Fisher’s exact test. All statistical tests were two-tailed tests with a significance level of *P* < 0.05. Data analysis was performed on IBM SPSS 20.0 software (IBM Corp., Armonk, NY, USA).

### Ethics and permits

The experiments comply with the current laws of China. Experimental procedures were in accordance with the Animal Research Ethics Committee of Hainan Provincial Education Centre for Ecology and Environment, Hainan Normal University (No. HNECEE-2012-004) and Experimental Animal Ethics Committee of Guizhou Normal University (No. 2021001).

## Results

**Fig. 2:**
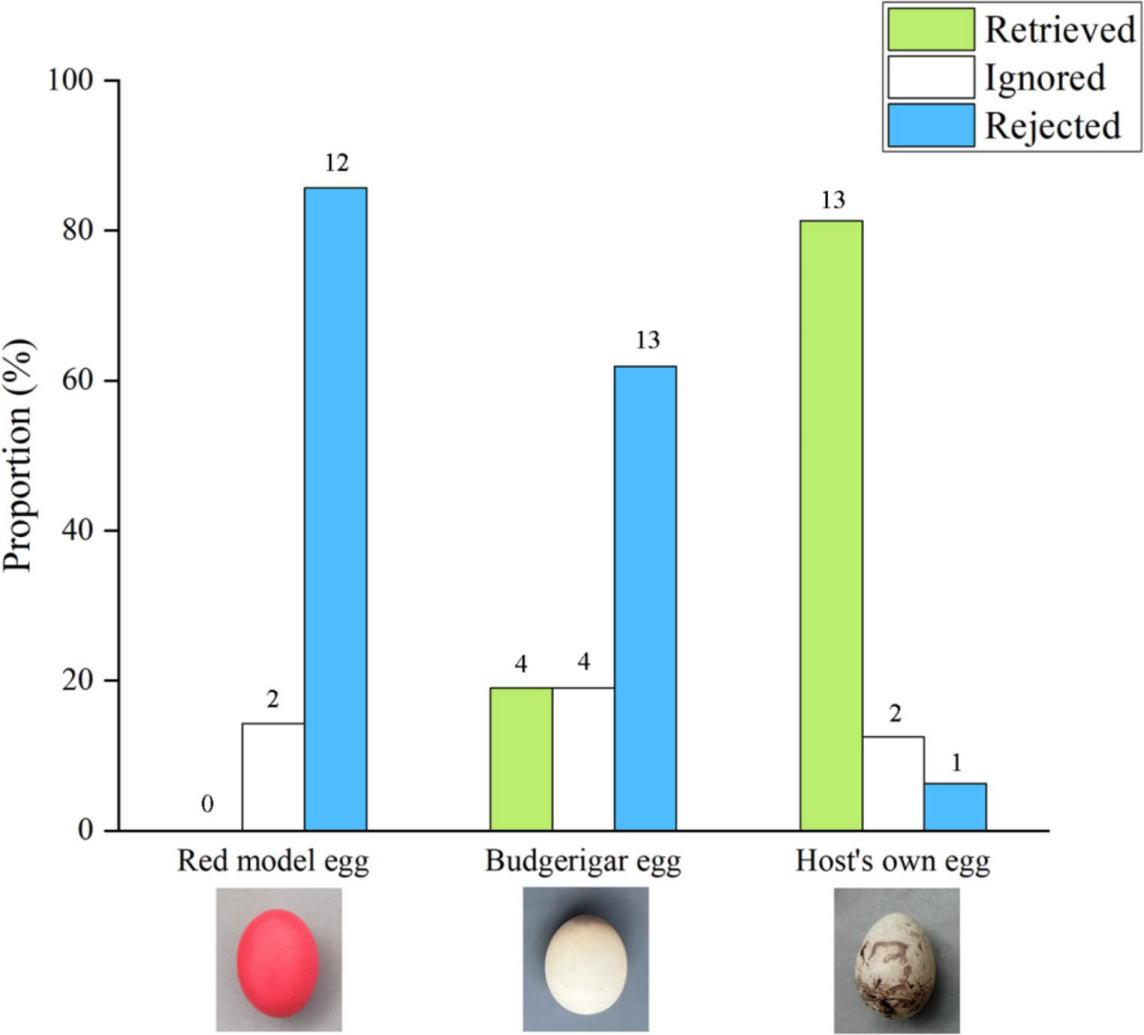
Comparison of retrieval, ignoring, and rejection rates of different types of eggs on the edge of the nest by south rock buntings

We conducted a total of 51 egg retrieval experiments on south rock buntings, of which 14 were with red model eggs, 21 with budgerigar eggs, and 16 with the host’s own eggs. There was a significant difference in the retrieval behavior of the south rock bunting for red model eggs, budgerigar eggs, and the host’s own eggs (0%, 19%, and 81.3%, respectively; Chi-squared test, *χ*^2^ = 25.46, *P* < 0.001; Fig. 2). Among them, there was no difference in host retrieval of the red model and budgerigar eggs (Fisher’s exact test, *P* = 0.133), and both were significantly lower than host retrieval of its eggs (Fisher’s exact test, all *P <* 0.05). There were also significant differences in rejection rates for red model eggs, budgerigar eggs, and the host’s eggs (85.7% vs. 61.9% vs. 6.3%, respectively, chi-squared test, *χ*^2^ = 20.572, *P* < 0.001; Fig. 2). Among them, the host’s rejection of the red model and budgerigar eggs did not differ (Fisher’s exact test, *P =* 0.252) and both were significantly higher than the host’s rejection of its own eggs (Fisher’s exact test, all *P <* 0.05).

**Fig. 3:**
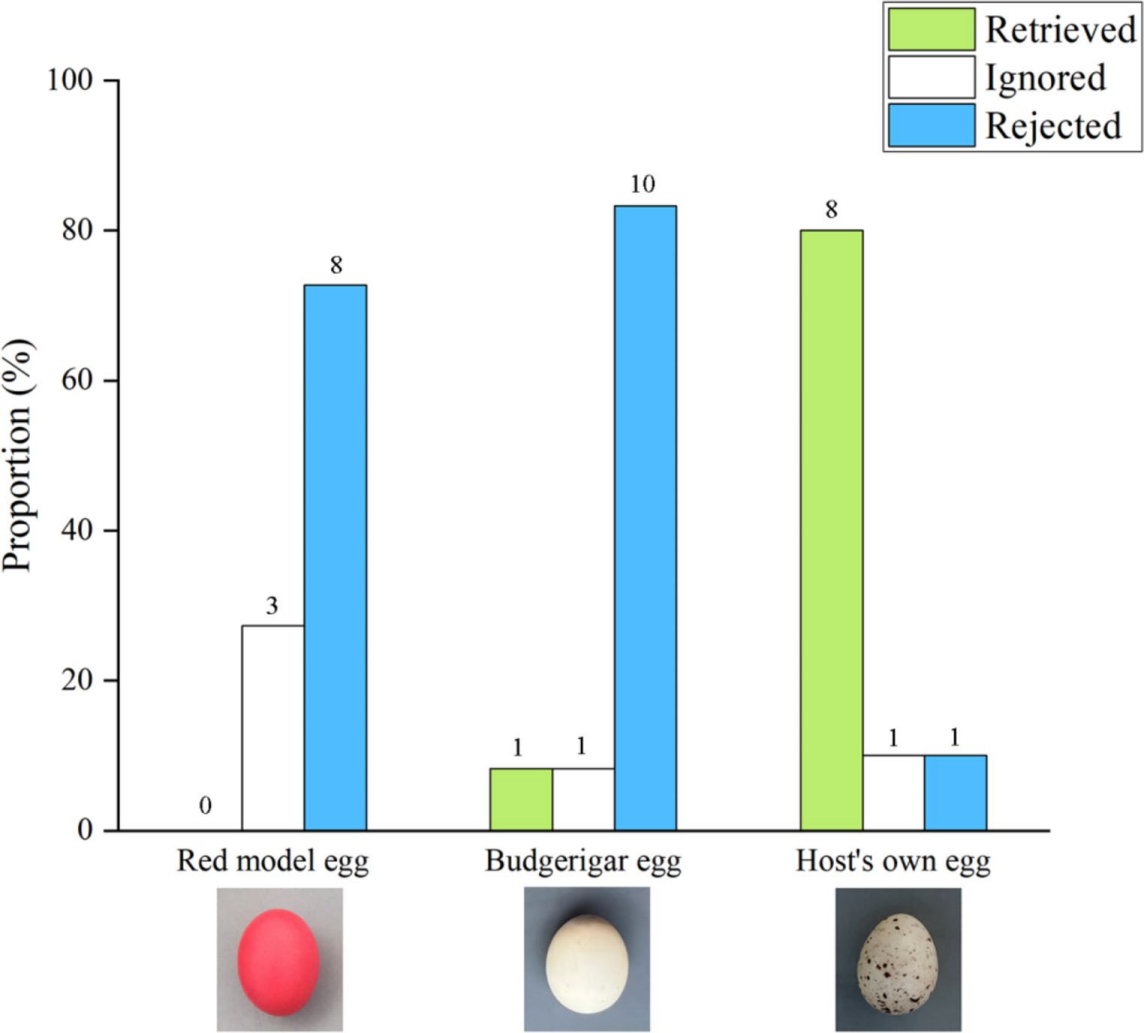
Comparison of retrieval, ignoring, and rejection rates of different types of eggs on the edge of the nest by yellow-throated buntings

We conducted a total of 33 egg retrieval experiments on yellow-throated buntings, of which 11 were with red model eggs, 12 with budgerigar eggs, and 10 with the host’s own eggs. There was also a significant difference in the retrieval behavior of the yellow-throated bunting for red model eggs, budgerigar eggs, and the host’s own eggs (0% vs. 8.3% vs. 80%, respectively; Fisher’s exact test, *χ*^2^ = 18.224, *P* < 0.001; Fig. 3). Among them, there was no difference in host retrieval of red model and budgerigar eggs (Fisher’s exact test, *P* = 1.000), and both were significantly lower than host’s retrieval of its own eggs (Fisher’s exact test, all *P <* 0.05). There were also significant differences in rejection rates for red model eggs, budgerigar eggs, and the host’s eggs (72.7% vs. 83.3% vs. 10%, Fisher’s exact test, *χ*^2^ = 13.305, *P* = 0.001; Fig. 3). Among them, the host rejection of the red model and budgerigar eggs did not differ (Fisher’s exact test, *P =* 0.640) and both were significantly higher than the host rejection of its eggs (Fisher’s exact test, all *P <* 0.05).

## Discussion

Testing host responses to different types of eggs on the edge of the nest can help study the interrelationships and coevolutionary arms race between avian brood parasites and their hosts. Results from this study were consistent with our hypothesis that the south rock bunting and yellow-throated bunting would be able to recognize and differentiate between non-mimetic eggs and their own eggs at the nest edge and would be able to retrieve their own eggs and reject most non-mimetic eggs. In particular, both the south rock bunting and yellow-throated bunting did not retrieve any completely non-mimetic red model eggs. Apart from the yellow-throated bunting having a slightly lower rejection rate for the red model egg than for the budgerigar egg, the results of the other experimental groups show that as the mimicry of the eggs at the nest edge increases, the host’s rate of egg retrieval increases and the rejection rate decreases.

This result was similar to that of the study conducted by Yang et al. (2019, Yang and Zhang 2024) but was inconsistent with that of Lyon and Shizuka (2017). The act of egg retrieval is thought to be primitive and instinctive as a result of convergent evolution in birds (Poulsen 1953; Lank, 1991). Unlike ground-nesting precocial birds, however, some cuckoo hosts may have the ability to differentiate and reject foreign eggs that are not their own while maintaining the instinct to retrieve them (Yang et al. 2019; Liu et al. 2023). The risk of brood parasitism and the degree of egg mimicry affect host thresholds for egg rejection (Davies et al. 1996; Svennungsen and Holen 2010; Soler 2017). Correspondingly, these might also be important factors influencing the motivation of parent birds to retrieve eggs, as the experimental results suggest that visual cognition processes by parent birds in retrieving or rejecting eggs at the nest edge should be consistent with that used in accepting or rejecting eggs inside the nest. Previous studies have shown that these two bunting species are rarely parasitized and are potential hosts for the cuckoo (Yang et al. 2012; Zhang et al. 2023). However, these populations have strong recognition and rejection of non-mimetic eggs. This is probably because they have evolved strong egg recognition abilities that have been retained due to a history of high parasitism with cuckoos (Honza et al. 2004; Yang et al. 2014; Medina and Langmore 2015). The fact that these two bunting species can directly decide to retrieve or reject eggs at the nest edge without needing to place them in the nest for further comparison with their eggs suggests that their ability to identify and distinguish between their eggs and foreign eggs is likely based on a template recognition mechanism, whereby imprinting on one’s own eggs serves as a template for accurately recognizing foreign eggs. The risk of nest predation may also be an important factor influencing egg retrieval by parent birds. Nest predation is a major factor contributing to reproductive failure in birds, and it poses a significant risk to the survival and safety of the parent birds themselves (Martin 1992; Lima 2009; Ibáñez-Álamo et al. 2015). Therefore, it is a major factor influencing the life history and regulating the behavior of birds (Martin 1992; Schmidt and Whelan 1999; Lima 2009; Ibáñez-Álamo et al. 2015). Nest sanitation behavior in birds is also thought to be a primitive instinctive behavior, objects such as feces and eggshells in the nest may be cues by predators searching for the nest, so keeping the nest clean helps to reduce the risk of detection by predators (Tinbergen et al. 1962; Petit et al. 1989). Correspondingly, eggs or other objects at the nest edge may be perceived by parent birds as attractants to nest predators, thus potentially leading to a higher risk of nest predation. Ground nests are inherently at higher risk of predation compared to other types of nests (Manolis et al. 2002; Roos et al. 2018; Minias and Janiszewski 2023), thus timely retrieval of eggs at the nest edge by the parent bird or carrying the eggs away is necessary for ground-nesting birds.

In a previous study, the south rock bunting and yellow-throated bunting were found to reject 76.9 and 82.4% of red model eggs in the nest, respectively (Zhang et al. 2023). This result aligns with the findings of this study, reflecting the rejection rate of red model eggs placed at the edge of the nest by both the bunting species. This also suggests that egg position does not affect host decision-making, whereas Yang et al. (2019) revealed green-backed tits to be more sensitive to eggs outside the nest. The relative frequency of events under natural conditions determines species’ behavior and strategy. Buntings have open-ground nests (Ding et al. 2019), and the probability of an event in which a cuckoo lays a parasitic egg on the edge of its nest is lower in comparison to a cavity nest in a confined space. Therefore, compared to the green-backed tit, which builds nests in the cavity, buntings that breed in open-ground nests should not be more sensitive to eggs at the nest edge than to eggs inside the nest.

Few individuals made an “incorrect” decision. In the budgerigar egg experiment, a few individuals retrieved the foreign eggs. In the experiment with their own eggs, several individuals also did not retrieve the eggs, and several neither chose to retrieve nor reject the eggs. This may result from differences in age and experience of individuals within the population. Some host recognition and rejection of foreign eggs have been found to correlate with age (Lotem et al. 1992; Molina-Morales et al. 2014; Martínez et al. 2020), with older or more experienced parents recognizing and rejecting parasitized eggs more quickly or accurately. Thus, the juvenile and inexperienced parental birds in this study may lack the experience to make decisions and choose to ignore them or make poor decisions when confronted with eggs on the edge of the nest. However, we did not examine the age of birds in this study due to the limited population of both bunting species within the study area, the inclination for nests to be deserted due to the capture of parents during the incubation phase, and the elevated risk of predation on ground nests.

In summary, we examined the egg retrieval behavior of two sympatric breeding bunting hosts to various mimicry of eggs. Both the south rock bunting and yellow-throated bunting accurately identified and distinguished between highly non-mimetic eggs and their own eggs at the nest edge, and they mostly chose to retrieve their eggs and reject non-mimetic eggs. This is consistent with their response to eggs in the nest, suggesting that change in egg position does not affect the behavior of the parent birds. Nest predation pressure and the risk of brood parasitism may be the main factors driving and regulating host perceptions and decision-making regarding different eggs at the nest, whereas age and experience may be responsible for behavioral differences among individuals within populations. Regarding specific clues they use to identify and make decisions about eggs at the nest edge, further research should be conducted on gradient quantification in terms of egg size, speckles, and egg color, among others.

## Electronic supplementary material

Below is the link to the electronic supplementary material.

Supplementary Material 1

## Data Availability

No datasets were generated or analysed during the current study.
